# Letermovir Administration to Prevent Cytomegalovirus Reactivation Is the Potential Risk of Chronic Graft-*Versus*-Host Disease in Patients Who Received Haploidentical Stem-Cell Transplantation With Post-Transplant Cyclophosphamide

**DOI:** 10.3389/fonc.2021.666774

**Published:** 2021-04-29

**Authors:** Toshiki Terao, Ken-ichi Matsuoka, Kentaro Narita, Takafumi Tsushima, Satoshi Yuyama, Ayumi Kuzume, Rikako Tabata, Daisuke Miura, Masami Takeuchi, Kosei Matsue

**Affiliations:** ^1^ Division of Hematology/Oncology, Department of Internal Medicine, Kameda Medical Center, Chiba, Japan; ^2^ Department of Hematology and Oncology, Okayama University Graduate School of Medicine, Dentistry and Pharmaceutical Sciences, Okayama, Japan; ^3^ Department of Pharmacy, Kameda Medical Center, Chiba, Japan

**Keywords:** letermovir, chronic graft-*versus*-host disease, cytomegalovirus, haploidentical stem-cell transplantation, post-transplant cyclophosphamide, HLA-DR+ activated T-cell, lymphocyte recovery

## Abstract

The prevention of chronic graft-*versus*-host disease (cGVHD) is important for recipients of hematopoietic stem-cell transplantation (HSCT). As one of the etiologies, the relationship between early T-cell recovery and subsequent cGVHD development has been the focus of attention. Recently, letermovir (LTV) was approved for preventing cytomegalovirus (CMV) reactivation in the early transplantation phase. Although CMV affects the immune reconstitution after HSCT, the impacts of LTV to prevent CMV reactivation on early T-cell recovery and cGVHD have not been fully investigated. We aimed to identify early T-cell recovery under LTV at day 30 in 15 and 33 recipients from matched related donors (MRDs) and haploidentical donors with post-transplant cyclophosphamide (PTCy-haplo), respectively. Early increases in the levels of total lymphocytes and HLA-DR^+^ activated T-cells at day 30 were observed under CMV prophylaxis by LTV only in PTCy-haplo recipients and not in MRD recipients. Moreover, PTCy-haplo recipients with LTV showed a significantly higher incidence of cGVHD, but not acute GVHD. Our observations suggest that an early increase in the levels of HLA-DR^+^ activated T-cells may be implicated in the development of cGVHD in patients treated with PTCy who received LTV. Further studies are warranted to validate our results and elucidate the detailed mechanisms of our new insights.

## Introduction

Recently, it has been reported that *in vivo* T-cell depletion therapy with antithymocyte globulin (ATG) or post-transplant cyclophosphamide (PTCy) is associated with the suppression of subsequent development of chronic graft-*versus*-host disease (cGVHD), suggesting the importance of regulating the early T-cell recovery after hematopoietic stem cell transplantation (HSCT) for the long-term immune tolerance (1, 2). Although the kinetics of lymphocyte recovery and its correlation with post-HSCT outcomes are well-established in the setting of matched related donors (MRDs), there are relatively few data obtained in the setting of haploidentical donors and post-transplant cyclophosphamide use (PTCy-haplo) (3). Cytomegalovirus (CMV) reactivation is an important cause of morbidity and mortality after allogeneic HSCT, which has also been reported to affect both early and long-term immune reconstitution (4). Moreover, PTCy-haplo transplant recipients show a high rate of CMV reactivation (about 70%) early after transplantation (5–7). Therefore, theoretically, a new anti-CMV prophylaxis agent letermovir (LTV) approved in 2018 in some countries including Japan could be considered for a subset of PTCy-haplo patients (8, 9). However, because LTV has been approved only recently, there is still a paucity of literature on its use for the prevention of lymphocyte recovery caused by CMV reactivation in PTCy-haplo transplant recipients (10).

In this study, we aimed to examine the early HLA-DR^+^ activated T-cell recovery in MRDs and PTCy-haplo transplant recipients treated with LTV for CMV prophylaxis and determine the association of lymphocyte recovery with cGVHD development. All participants or their family members provided written informed consent for inclusion in retrospective studies. This study was conducted in accordance with the Declaration of Helsinki and was approved by the ethical review board of the Kameda Medical Center.

## Methods

We retrospectively analyzed 15 MRD and 33 PTCy-haplo transplant recipients who received allogeneic HSCT as grafts of peripheral blood (PB) at our center from January 1, 2014 to August 31, 2020 (**Table 1**). The observation period ended on November 31, 2020. The haploidentical donor was defined as a relative who had two or more mismatches in human leukocyte antigen (HLA)-A, -B, -C, and -DRB1 alleles. GVHD prophylaxis was performed as follows: high-dose cyclophosphamide (50 mg/kg) on days 3 and 4, and both tacrolimus and mycophenolate mofetil (MMF) from day 5 in PTCy-haplo transplant recipients, and short-term methotrexate on day 1, 3, and 6 or MMF and calcineurin inhibitors from day -1 in MRD transplant recipients. CMV reactivation was defined as the detection of 3 or more positive cells per 50,000 cells by pp65 CMV-antigenemia assay in patients’ peripheral blood without obvious end-organ dysfunction, monitored routinely weekly until day 100 or as long as clinically indicated unnecessary. CMV disease was defined by end-organ dysfunction attributable to CMV confirmed by organ biopsy because these conditions would require the administration of anti-CMV drugs (11, 12).

**Table 1:** Patients’ baseline characteristics.

Characteristics	MRD			PTCy-haplo		
	LTV+	LTV-		LTV+	LTV-	
	n = 8	n = 7	*p* value	n = 17	n = 16	*p* value
Patient age at transplant (median, range)	55 (20, 60)	57 (36, 69)	0.33	55 (17, 68)	57 (20, 68)	0.69
Sex (male, %)	6 (75.0)	6 (85.7)	1	14 (82.4)	11 (68.8)	0.44
Diagnosis (n, %)			1			0.66
AML/MDS	3 (37.5)	4 (57.1)		6 (35.3)	9 (56.2)	
ALL	2 (25.0)	1 (14.3)		2 (11.8)	1 (6.2)	
ML	1 (12.5)	0		5 (29.4)	2 (12.5)	
MM/PCL	1 (12.5)	1 (14.3)		2 (11.8)	1 (6.2)	
others	1 (12.5)	1 (14.3)		2 (11.8)	3 (18.8)	
Disease status (n, %)			0.78			1
in any CR	3 (37.5)	4 (57.1)		5 (29.4)	4 (25.0)	
not CR	4 (50.0)	2 (28.6)		10 (58.8)	10 (62.5)	
other	1 (12.5)	1 (14.3)		2 (11.8)	2 (12.5)	
DRI (high/very high, %)^†^	4 (50.0)	2 (28.6)	0.61	10 (66.7)	8 (57.1)	0.71
MAC vs. RIC (RIC, %)	3 (37.5)	5 (71.4)	0.32	12 (70.6)	10 (62.5)	0.72
ECOG PS (<2, %)	5 (62.5)	6 (85.7)	0.57	11 (64.7)	11 (68.8)	1
CMV serostatus (n, %)^††^			1			0.82
D-/R+	1 (25.0)	0		4 (33.3)	5 (50.0)	
D+/R-	0	0		1 (8.3)	0	
D+/R+	3 (75.0)	2 (100)		7 (58.3)	5 (50.0)	
CMV reactivation (+, %)	1 (12.5)	4 (57.1)	0.11	3 (17.6)	13 (81.2)	<0.001
ABO match (n, %)			0.71			0.74
Matched	7 (87.5)	5 (71.4)		9 (52.9)	7 (43.8)	
Major mismatch	1 (12.5)	1 (14.3)		3 (17.6)	5 (31.2)	
Minor mismatch	0	1 (14.3)		5 (29.4)	4 (25.0)	
Infusion CD34+ cells (×10^6^/kg) (median, range)	3.6 (2.0, 5.4)	3.6 (1.8, 6.1)	0.91	4.2 (2.0, 10.4)	3.7 (1.7, 5.0)	0.18
Prophylaxis of GVHD			0.28			1
PTCy + Tac + MMF	0	0		17 (100)	16 (100)	
short MTX + CNI	7 (87.5)	4 (57.1)		0	0	
MMF + CNI	1 (12.5)	3 (42.9)		0	0	
Donor age (median, range)	53 (33, 61)	54 (30, 60)	0.9	29 (15, 55)	34 (20, 59)	0.1
Donor type (n, %)			0.32			0.84
Children	1 (12.5)	0		13 (76.5)	11 (68.8)	
Parents	2 (25.0)	0		1 (5.9)	1 (6.2)	
Siblings	5 (62.5)	7 (100)		3 (17.6)	4 (25.0)	
HLA match (n, %)			NA			0.077
4/8	0	0		12 (70.6)	6 (37.5)	
5/8	0	0		5 (29.4)	7 (43.8)	
6/8	0	0		0	3 (18.8)	
aGVHD, grade II-IV (n, %)	1 (12.5)	3 (42.9)	0.28	6 (35.3)	8 (50.0)	0.49
Additional immunosuppression^*^before day 30^†††^	3 (42.9)	2 (28.6)	1	4 (28.6)	7 (43.8)	0.47

AML, acute myeloid leukemia; ALL, acute lymphoblastic leukemia; CMV, cytomegalovirus; CNI, calcineurin inhibitor; CR, complete remission; DRI, disease risk index; ECOG PS, Eastern Cooperative Oncology Group Performance Status; GVHD, graft-versus-host disease; HLA, human leukocyte antigen; LTV, letermovir; MAC, myeloablative conditioning; MDS, myelodysplastic syndromes; ML, malignant lymphoma; MM, multiple myeloma; MMF, mycophenolate mofetil; MRD, matched-related donor; MTX, methotrexate; NA, not applicable; PCL, plasma cell leukemia; PTCy, post-transplant cyclophosphamide; RIC, reduced-intensity conditioning; Tac, tacrolimus.

^†^n = 13 and 29.

^††^n = 6 and 22.

^†††^n = 14 and 30.

*Additional immunosuppression indicates additional systemic prednisolone or methylprednisolone initiation before day 30 for acute GVHD or engraftment syndrome.

cGVHD diagnosis and grading were based on a previous report (13). Relapse-free survival (RFS) was defined as the time between transplantation and relapse, death, or the end of the study period. Overall survival (OS) was defined as the time between transplantation and death or the end of the study period. The probability of RFS and OS was estimated using the log-rank test. Competing events for cGVHD were death or relapse without GVHD. The groups were compared using Gray’s test. All statistical analysis was conducted using R version 3.1.2 (The R Foundation for Statistical Computing, Vienna, Austria) and using the EZR software package (Saitama Medical Center, Jichi Medical University, Shimotsuke, Japan), which is a graphical user interface for R (14).

## Results

Baseline clinical characteristics of the patients who received PTCy-haplo or MRDs are summarized in **Table 1**. The median age at transplantation in MRD and PTCy-haplo was both 56-year-old and 12 and 25 recipients in MRD and PTCy-haplo, respectively, were male. The background hematologic malignancies were acute myeloid leukemia/myelodysplastic disorders (AML/MDS) in 7, acute lymphoblastic leukemia (ALL) in 3, and malignant lymphoma (ML) in 1 in MRD recipients, and AML/MDS in 15, ALL in 3, and 7 in ML in PTCy-haplo recipients. Since LTV was approved in Japan in 2018, patients with LTV received transplantation after 2018. Overall, the patients’ backgrounds were similar between before and after LTV administration. The RFS of MRD (n=13) and PTCy-haplo (n=28) transplant recipients at 15 months was 75.5% and 55.5% (95% confidence interval [CI]: 42.6-91.4% and 31.9-73.8%), respectively. The OS of MRD and PTCy-haplo at 15 months was 66.7% and 55.1% (95% CI: 37.5–84.6% and 36.0–70.6%), respectively. The RFS and OS were not significantly different in terms of CMV reactivation and disease.

**Figure 1:**
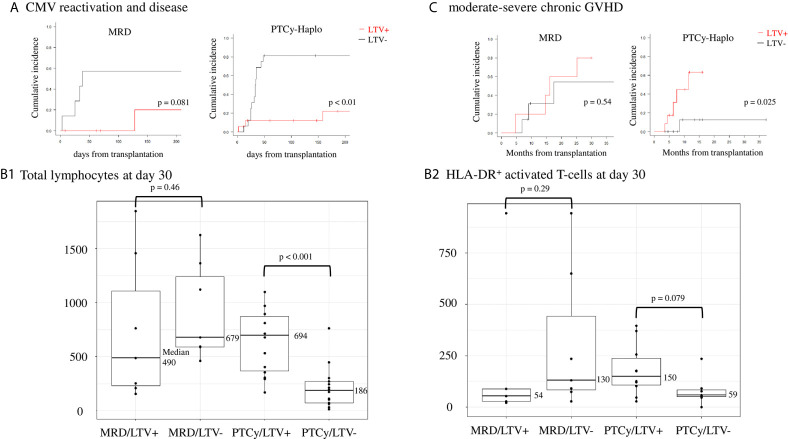
Effects of CMV prophylaxis with LTV in matched related donor (MRD) transplantation and haploidentical transplantation with post-transplant cyclophosphamide (PTCy-haplo). **(A)** CMV reactivation and disease rate at day 100 in MRD and PTCy-haplo transplant recipients. Statistical analysis was performed using the Kaplan−Meier method with log-rank analysis and Gray’s test. **(B)** Counts of total lymphocytes (left, **B1**) and HLA-DR^+^ activated T-cells (right, **B2**) in MRD and PTCy-haplo transplant recipients treated or not with LTV. Statistical analysis was performed using the Mann−Whitney U test. **(C)** Development of moderate-to-severe chronic GVHD at 15 months in MRD and PTCy-haplo patients.

Regarding the efficacy of LTV, MRD transplant recipients prophylactically treated with LTV had a lower CMV reactivation and disease rate on day 100 than those not treated (0% *vs*. 57.1%, 95% CI: 0–0% *vs*. 26.6–90.2%; *p* = 0.081). Similarly, LTV-treated PTCy-haplo patients showed a significantly lower rate of CMV reactivation and disease on day 100 than untreated patients (12.2% *vs*. 81.2%, 95% CI: 3.4–40.5% *vs*. 59.8–95.4%; *p* = 0.001) (**Figure 1A**). Two PTCy-haplo recipients had CMV-disease (both CMV-colitis), and these patients was survived by ganciclovir treatment. No CMV-disease occurred in MRD recipients.

Next, we examined early T-cell recovery on day 30 in MRD and PTCy-haplo patients (**Table 2**). The median total lymphocyte recovery was delayed in the PTCy-haplo group compared with the MRD group (298/μL *vs*. 636/μL, *p* = 0.015). To investigate the effect of CMV prophylaxis by LTV on T-cell recovery, we further divided MRD and PTCy-haplo patients into LTV-treated and -untreated subgroups (**Table 1**); the patient backgrounds in each of the two MRD or PTCy-haplo subgroups were almost compatible. On day 30, there was no significant difference in total lymphocyte counts between LTV-treated and untreated MRD patients; however, a significant increase in total lymphocyte count was observed in LTV-treated PTCy-haplo patients (median 694/μL *vs*. 186/μL in the untreated group; *p* < 0.001) (**Figure 1B1**). We also detected an increasing trend to increase in the levels of CD3^+^HLA-DR^+^, CD4, and CD8 T-cells in the LTV-treated compared with the LTV-untreated PTCy-haplo patients (median HLA-DR^+^ activated T-cells:150/μL *vs*. 59/μL, *p* = 0.079, CD4^+^ and CD8^+^ T-cells are shown in **Table 2**). However, there was no statistically significant difference in HLA-DR^+^ activated T-cells, CD4^+^, and CD8^+^ T-cells between LTV-treated and -untreated MRD transplant recipients (median HLA-DR^+^ activated T-cells: 54/μL *vs*. 130/μL, *p* = 0.29) on day 30 (**Figure 1B2**, **Table 2**).

**Table 2:** Lymphocytes count in MRD and PTCy-haplo.

median, μL (range)	N	MRD				PTCy-haplo		
		LTV+	LTV-	*p* value	N	LTV+	LTV-	*p* value
day 30								
Total lymphocytes	14	490 (156, 1848)	679 (460, 1625)	0.46	30	694 (168, 1100)	186 (15, 760)	<0.001
CD3^+^	12	203 (148, 1533)	441 (276, 1173)	0.27	17	275 (58, 520)	102 (4, 302)	0.019
CD4^+^	12	129 (92, 346)	170 (163, 346)	0.53	17	84 (15, 232)	38 (2, 83)	0.032
CD8^+^	12	84 (42, 924)	148 (64, 982)	0.2	17	148 (31, 406)	48 (1, 205)	0.055
CD19^+^	12	0 (0, 18)	0 (0, 11)	1	17	0 (0, 8)	0 (0, 0)	0.26
CD56^+^	12	37 (4, 203)	190 (78, 243)	0.034	17	125 (20, 388)	81 (1, 425)	0.097
CD3^+^HLA-DR^+^	12	54 (22, 942)	130 (29, 941)	0.29	17	150 (29, 396)	59 (1, 235)	0.079

As the early recovery of HLA-DR^+^ activated T-cells was observed only in PTCy-haplo patients prophylactically receiving LTV, we further investigated the rate of moderate to severe cGVHD based on 3.3 months landmark analysis. PTCy-haplo transplant recipients treated with LTV showed a significant increase in the cGVHD rate at 15 months compared with those not treated (63.3% *vs*. 12.5%, 95% CI: 30.5–93.7% *vs*. 1.9–61.3%, *p* = 0.025; **Figure 1C**). However, no difference in the cGVHD rate was observed among MRD patients treated or not with LTV. The cumulative incidence of grade II-IV acute GVHD (aGVHD) was not significantly different in patients with LTV and without LTV in both MRD and PTCy-cohort (**Table 1**).

## Discussion

These data demonstrated that only PTCy-haplo but not MRD transplant recipients subjected to CMV prophylaxis by LTV showed early recovery of total lymphocytes as well as HLA-DR^+^ activated T-cells. These PTCy-haplo patients with LTV showed a significantly high incidence of cGVHD, but not aGVHD in this study. In general, a significant increase of acute and chronic GVHD has been reported in recipients using graft from PB which content high HLA-DR^+^ activated T-cells (15–17). However, due to the protective effect of PTCy on regulatory T-cells (18, 19), it is considered that the incidence of aGVHD was not increased, which was compatible to the previous report (9). On the other hand, although the reason for early HLA-DR^+^ activated T-cell expansion observed after CMV prophylaxis by LTV only in PTCy-haplo patients is still unknown, possible underlying mechanisms could include shifts in cytokine dynamics for CMV protection and changes in the integrity and heterogeneity of the T-cell repertoire (20–22).

The risk factors reported for cGVHD development in PTCy-haplo transplant recipients, including reduced-intensity conditioning regimens, older donor age, and PB as a graft source (23), are associated with increased alloreactive T-cell proliferation and exhaustion. T-cell-depleting antibodies such as ATG can suppress the development of cGVHD by removing early alloreactive T-cells (1). The other possible mechanism of increased cGVHD in PTCy-haplo recipients with LTV was insufficient T-cell suppression in the early-phase of transplantation. Therefore, to inhibit early T-cell expansion and prevent cGVHD in PTCy-haplo transplant recipients, additional prolonged immunosuppression after PTCy administration could be considered.

The limitations of our study include the heterogeneous patient background and small sample size. The data on T-cells after day 30 and functional assay for mediating alloreactive T-cells such as interferon-gamma and tumor necrosis factor-alfa were not collected systematically. The correlation between HLA-DR+ activated T-cells and chronic but not acute GVHD might seem intriguing, however, we are unable to throw further light on the mechanistic pathways behind this association in the absence of longitudinal data. Further prospective studies on the relationship between detailed T-cell analysis and cGVHD under LTV are warranted because the use of LTV is expanding in the clinical practice. Despite these limitations, the uniformity of transplantation grafts (PB from haploidentical relatives) and GVHD prophylaxis (high-dose Cy, then tacrolimus and MMF) in PTCy-haplo patients could be considered a strength of this study.

In conclusion, our results revealed early HLA-DR^+^ activated T-cell expansion in PTCy-haplo but not in MRD patients who received LTV for CMV prophylaxis. These LTV-treated PTCy-haplo recipients showed a higher incidence of cGVHD; thus, these patients might be subjected to prolonged immunosuppression to prevent cGVHD development. Further studies are warranted to validate our findings and elucidate the detailed mechanisms underlying the effects reported here.

## Data Availability Statement

The raw data supporting the conclusions of this article will be made available by the authors, without undue reservation.

## Ethics Statement

This study was conducted in accordance with the Declaration of Helsinki and was approved by the ethical review board of the Kameda Medical Center. Written informed consent to participate in this study was provided by the participants’ legal guardian/next of kin.

## Author Contributions

TTe conceived, designed, and initiated the study, acquired data, and wrote the manuscript. K-iM, KN, TTs, and SY participated inwriting the manuscript. TTe and KN performed statistical analyses. TTe, KN, TTs, AK, RT, DM, MT, and KM provided patient care. KM supervised the study. All authors have reviewed and approved the final manuscript and agreed to be accountable for all aspects of the work in ensuring that questions related to the accuracy or integrity of any part of the work are appropriately investigated and resolved. All authors contributed to the article and approved the submitted version.

## Conflict of Interest

The authors declare that the research was conducted in the absence of any commercial or financial relationships that could be construed as a potential conflict of interest.
